# Transcriptome-based network analysis of cell cycle-related genes in response to blue and red light in maize

**DOI:** 10.1093/aobpla/plad079

**Published:** 2023-12-11

**Authors:** Tiedong Liu, Xiwen Zhang, Shengqun Liu

**Affiliations:** College of Agriculture, Fujian Agriculture and Forestry University, Shang Xia Dian Road 15#, Cangshan District, Fuzhou 350002, Fujian, China; Key Laboratory of Ministry of Education for Genetics, Breeding and Multiple Utilization of Crops, College of Agriculture, Fujian Agriculture and Forestry University, Shang Xia Dian Road 15#, Cangshan District, Fuzhou 350002, Fujian, China; College of Mechanical and Electronic Engineering, Fujian Agriculture and Forestry University, Shang Xia Dian Road 15#, Cangshan District, Fuzhou 350002, Fujian, China; Northeast Institute of Geography and Agroecology, Chinese Academy of Sciences, No. 4888, Shengbei Street, Gaoxin North District, Changchun 130000, Jilin, China

**Keywords:** Cell cycle, cell division, light regulation, transcriptome, *Zea mays* L

## Abstract

In maize, blue and red light are key environmental factors regulating cell-cycle progression. We used transcriptomics to investigate and compare differential gene expression under the four light conditions: red light, blue light, red converted to blue and blue converted to red. A total of 23 differentially expressed genes were identified. The gene–gene interaction analysis indicated a significant interaction between four unidentified genes, *100191551*, *pco143873*, *100284747* and *pco060490*, and cell-cycle-related genes. Using multiple sequence alignment analysis and protein structure comparisons, we show here that these four unidentified genes were characterized as *ALP1-like*, *ALP1*, *cyclin P1-1* and *AEBP2*, respectively. By constructing a protein–protein interaction network, we inferred that *100191551* and *pco143873* are potentially regulated to avoid DNA damage by abiotic stress response factors in the cell cycle. The gene *100284747* regulates the cell cycle in response to phosphate starvation signalling. The gene *pco060490* potentially negatively regulates the cell cycle through the mediation of Histone H3 and CYCD6 in response to red light. In conclusion, the cell-cycle-related genes are sensitive to blue and red light, and four novel functional genes may be involved in the cell cycle.

## Teaser

The cellular development of maize is regulated by light quality. Both typical red light and blue light play a role. Numerous genes and proteins are involved in these regulatory processes. This article introduces four potential functional genes related to light quality regulation and their associated proteins. Validation was conducted using molecular biology methods.

## Introduction

The function of cell-cycle-related genes is accurately regulated by light signalling. Light-dependent onset of the cell cycle in plants is important and common (Huysman *et al.* 2013b). Cell proliferation and differentiation into different cell types depend on the specified stimulation. Cell division involves the division of one cell into two genetically identical daughter cells, and is tightly controlled during differentiation processes. During the cell-division process, gene transcription must be initially terminated and then reactivated once cell division is complete (Taguchi and Turki 2021). DNA replication determines the timing of mitosis. Specified cells often exit a mitotic cell cycle mode and switch to an endoreplication program in which DNA replication is continued without subsequent cell division (Lemmens *et al.* 2018; Wang *et al.* 2021). Faithful DNA replication requires that each origin of replication occurs only once per cell cycle. Therefore, cell division and DNA replication rely on the coordinated function of cell-cycle genes (De Veylder *et al.* 2002), and cell-cycle times are tightly associated with terminal-cell fates (Jakoby and Schnittger 2004). Red to far-red light accelerates the cell cycle among some microalgal species (Schulze *et al.* 2014), and UV–C light causes a variety of DNA lesions that block DNA replication (Painter and Howard 1982). DNA photolyase binds to the UV-induced lesion and uses blue light as a co-substrate to return the DNA to its undamaged state (Sancar 2003). Meanwhile blue light leads to bigger cells by delaying cell division as well as DNA synthesis compared to red light (Wagner *et al.* 2016). Yet note that specified cell differentiation may be regulated by complex signals (DongLiu *et al.* 2020).

The cell cycle is regulated by a lot of precise and complex mechanisms. These mechanisms depend on various proteins, such as cyclin-dependent kinases (CDK), cyclin, retinoblastoma protein (Rb), CDK inhibitors (ICK), *E2* promoter-binding factor (E2F)/dimerization partner (DP) transcription factors (E2F) and wee1 kinase protein (Wee1) (Frouin *et al.* 2005; Inzé and De Veylder 2006). The assembly of an extra copy of the cell is tightly mediated by a series of phosphorylation events driven by CDK. Cyclin-dependent kinases and cyclin bind a specific cyclin subunit to form a functional and active CDK–cyclin complex (Cheng *et al.* 2012). When Rb proteins are phosphorylated by CDK–cyclin complexes, cells exit the restriction (R) point and become committed to completing the cell cycle (Wang *et al.* 2013). CDK–cyclin complexes are regulated by a group of proteins known as ICK, which are degraded by Rb as a negative feedback mechanism (Zhao *et al.* 2012). In the Gap1 (G1) phase of the cell cycle, hypophosphorylated Rb sequesters the transcription factors E2F bound to their DP partners. Phosphorylation of the pocket protein by cyclin D- and cyclin E-dependent kinases 4/6 releases the E2F/DP complex. This process allows for transcriptional activity of E2F and progression of the cell through the S phase of the cell cycle (Sears and Nevins 2002). Central in regulating the transition between the Gap2 (G2) and mitosis (M) phases is Wee1, a tyrosine kinase. Wee1 negatively regulates entry into mitosis by phosphorylating the Tyr15 residue of CDK1, thus inactivating the CDK1/cyclinB complex and arresting the cell cycle (Magnussen *et al.* 2012). In maize, 54 cyclin genes, 16 CDK genes, 19 E2F genes, 12 ICK genes, 5 Rb genes and one Wee1 gene were reported (Xiao *et al.* 2021), but their characters and signalling networks have not yet been completely identified.

In our previous study, we evaluated the differential pattern of stomatal cell distribution in response to blue and red light in maize, and we concluded that the effects of light on cell division and specialized cell formation were more complicated than that reported previously (DongLiu *et al.* 2020; Liu and Zhang 2021a). Herein, we explore the regulatory networks of the cell cycle in maize at the level of the transcriptome under four light treatments (i.e. blue light (B), red light (R), blue converted to red light (BR) and red converted to blue light (RB)). We focus on the differential expression of cell-cycle-related genes under blue and red light conditions. Finally, we attempt to find novel functional genes that are involved in signalling pathways of the cell cycle in maize.

## Materials and Methods

### Plants and light conditions

The Xianyu335 cultivar of maize used in this study was obtained commercially. The formal identification of the plant material was undertaken by Tieling pioneer seed research company (Tieling, China). The plants are kept at the College of Agriculture, Fujian Agricultural and Forestry University, Fuzhou, China. The maternal lineage of Xianyu335 is PH6WC and the paternal lineage is PH4CV. Maize seedlings were cultivated in a climate-controlled cultivation chamber, designed and manufactured by the Center of Excellence for Research in Optoelectronic Agriculture at Fujian Agriculture and Forestry University. Uniform blue and red light-emitting diode lamps were distributed on the top of each chamber. Maize seedlings were cultivated under four light treatments: 450 nm blue (B), 660 nm red (R), 450 nm blue converted to 660 nm red (BR) and 660 nm red converted to 450 nm blue (RB). The lighting setting for BR was 72 h of blue light followed by 24 h of red light. The lighting setting for RB was 72 h of red light followed by 24 h of blue light. Light density was set at 150 μmol m^−2^ s^−1^. Forty-eight seedlings were cultivated in a light chamber for a 24-h photoperiod every day, and each treatment was repeated four times. The temperature and relative humidity of the cultivation environment were set at 25 °C and 70 %, respectively. Each fourth seedling leaf was collected for subsequent experiments.

### RNA sequencing and transcriptome analysis

Total RNA was extracted using the mirVana miRNA Isolation Kit (Ambion) following the manufacturer’s protocol. RNA integrity was evaluated using the Agilent 2100 Bioanalyzer (Agilent Technologies, Santa Clara, CA, USA). The samples with RNA Integrity Number ≥ 7 were subjected to subsequent analysis. The libraries were constructed using a TruSeq Stranded mRNA LTSample Prep Kit (Illumina, San Diego, CA, USA) according to the manufacturer’s instructions. Then these libraries were sequenced on the Illumina sequencing platform HiSeqTM 2500 (Illumina, San Diego, CA, USA) and 125bp/150bp paired-end reads were generated. A large amount of double-ended sequencing data was obtained on the Illumina platform. Clean RNA-seq reads from each sample were obtained using Trimmomatic software (Bolger *et al.* 2014). Then clean reads were mapped to the reference genome of maize B73_RefGen_v4. The hisat2 was used to sequence the clean reads with the reference genome for the position and characteristic information (Kim *et al.* 2015). Gene expression was quantified using the HTseq-count tool software (Anders *et al.* 2015). Differential expression analysis was performed using the DESeq software (Anders *et al.* 2012). *P* value < 0.05 and foldchange > 2 was set as the threshold for significantly differential expression. The DEGs were translated into proteins and then searched in BLASTP against related species to establish a predicted protein interaction network. The Cytoscape v 3.5.1 app MCODE was used to identify and extract densely connected sub-networks in the network (Shannon *et al.* 2003). Advanced options were 2° cutoff, 0.2 Node Score Cutoff and 5 K-Core.3.

### Data analysis and bioinformatics analysis

R toolkit was used to draw the pictures of the transcriptome analysis. The analysis of the phylogenetic tree was carried out using MEGA X (Kumar *et al.* 2018) and depicted using EvolView online tools (Subramanian *et al.* 2019). The signal network was depicted using Adobe Illustrator CC 2019 (Adobe Systems Incorporated, San Jose, CA, USA). Interaction networks of genes, proteins and metabolites were drawn using Cytoscape 3.5.1 (Shannon *et al.* 2003) and the method of Liu (Liu and Zhang 2021a). Conserved domains and protein motifs were analyzed using Pfam, MEME-CHIP, SMART and SCOP online tools (Machanick and Bailey 2011; Andreeva *et al.* 2019; Letunic *et al.* 2020; Mistry *et al.* 2020), and visualized using DOG2.0 (Ren *et al.* 2009). The Ensembl database was used to search the variation sites of unidentified differential expression genes (Howe *et al.* 2020). The secondary structure of unidentified proteins referring to the unidentified genes was predicted using online tools of PSIPRED 4.0 (http://bioinf.cs.ucl.ac.uk/psipred/). I-TASSER-MTD was used to identify the tertiary structural templates of the unidentified proteins (https://zhanggroup.org/I-TASSER-MTD/). Bar charts were plotted and statistical analyses were performed using GraphPad Prism 9.0 (GraphPad Prism Inc.). One-way ANOVA was used to analyse significant differences between the measured data by comparing their means. The significance level was set at 0.05 (*α*).

### qPCR

A total of 10 genes were randomly selected to perform qPCR. Total RNA was isolated using the RNAqueous® Total RNA Isolation Kit AM1912 (Life Technologies Corp., Grand Island, New York, USA). The RNA yield was determined using a NanoDrop 2000 Spectrophotometer (Thermo Scientific, Waltham, USA), and the integrity was evaluated using agarose gel electrophoresis with ethidium bromide stain. Quantified reactions were performed in a GeneAmp® PCR System 9700 (Applied Biosystems, Waltham, USA). RT-PCR was performed using a LightCycler® 480 II Real-time PCR Instrument (Roche, Basel, Switzerland) with QuantiFast® SYBR® Green qPCR Master Mix (Qiagen, Düsseldorf, Germany). Each sample was prepared in triplicate for analysis. The primer sequences were designed in the laboratory and synthesized by Generay Biotech (Generay, Shanghai, China) based on the mRNA sequences obtained from the NCBI database. ZmGAPDH was used as a housekeeping gene. The sequences of primers used in qPCR are listed in Supporting Information—Table S1. Genes were annotated using the Nr, Nt, Pfam, KOG/COG, Swiss-Prot, KO and GO databases, and then used in GO enrichment analysis and KEGG pathway analysis.

## Results

### The RNA-seq expression of cell-cycle-related genes in response to blue and red light in maize

**Figure 1.**
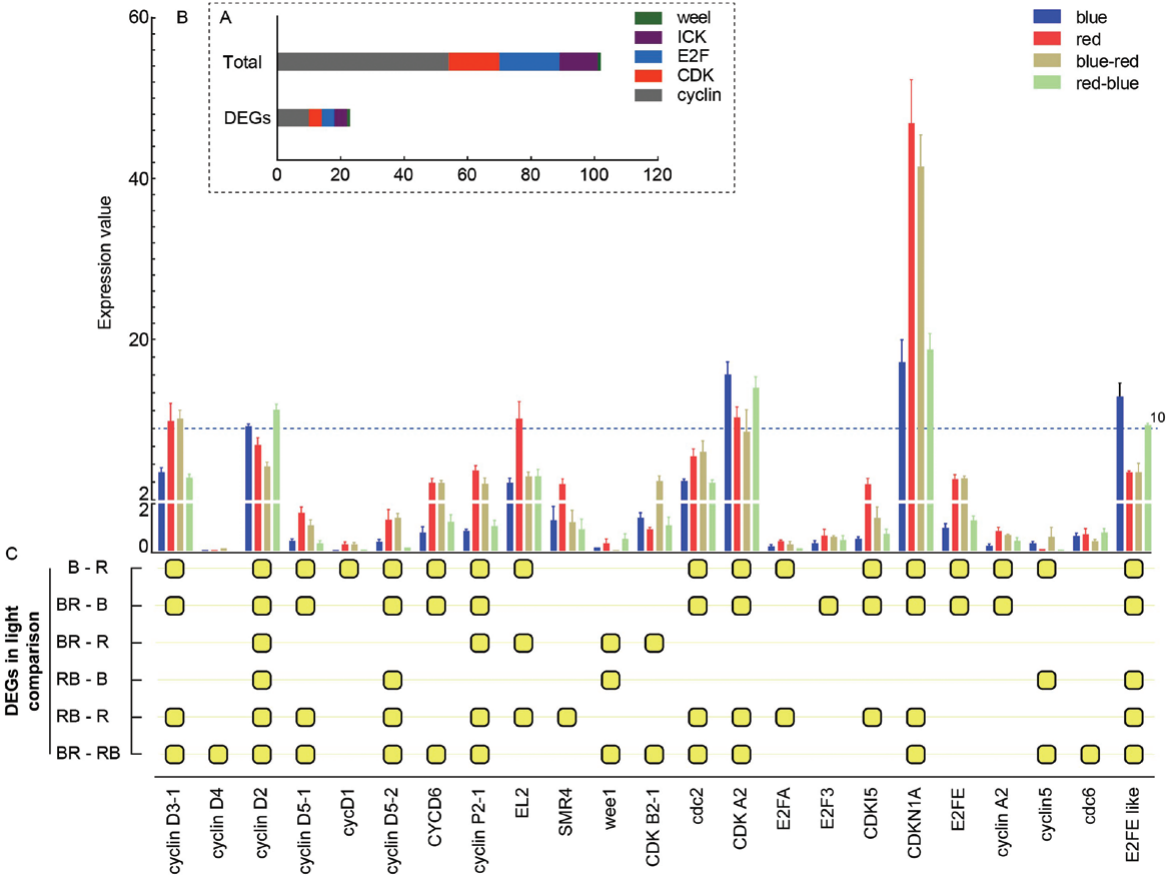
(A) The ratio of the number of DEGs in the total cell-cycle-related genes. (B) The RNA-seq expression of 23 DEGs of cell-cycle-related genes. (C) Comparison of 23 DEGs between six different groups among four light treatments.

Blue, red and blue-red light conversions have a significant influence on the transcriptional expression of cell-cycle-related genes. In this study, 10 of 54 cyclin genes, 4 of 16 CDK genes, 4 of 19 E2F genes, 4 of 12 ICK genes and 1 wee1 gene were identified as differentially expressed genes (DEGs) under one or more light treatments (Fig. 1A). Transcriptome profiling (Fig. 1B) indicated that the relative expression of six DEGs was significantly higher (higher than 10) than all other cell cycle DEGs, among which *cyclin D3-1* and *cyclin D2* belong to the cyclin family, *EL2*, *CDK A2* and *CDKN1A* belong to the ICK family, and *E2FE like* belongs to the E2F family. The overlapping and unique DEGs between various comparisons are clarified in Fig. 1C. C*yclin D3-1* and *CDKN1A* exhibited similar expression trends between light comparisons, and their relative expression under R and BR was significantly higher than under B and RB light. Under red light, the expression levels of *cyclin D3-1* were more than 2.83-fold and 4.21-fold higher than under B and RB, respectively. Under the BR treatment, the expression levels of *CDKN1A* were more than 2.89-fold and 4.30-fold higher than under B and RB, respectively. *Cyclin D3-1* limits the G1-to-S-phase transition (Menges *et al.* 2006). And *CDKN1A* is a gene that regulates cell-cycle progression, terminal differentiation and apoptosis (Wagner *et al.* 2001). *Cyclin D2*, *CDK A2* and *E2FE like* exhibited similar expression trends between light comparisons, and their relative expression under R and BR was significantly lower than under B and RB. Under RB light, the expression levels of *cyclin D2* were more than 1.22-fold, 1.63-fold and 2.67-fold higher than under B, R and RB, respectively. Under the B treatment, the expression levels of *CDK A2* were more than 1.52-fold, 1.87-fold and 1.11-fold higher than under R, BR and RB, respectively. Under B-light, the expression levels of *E2FE like* were more than 3.74-fold, 3.71-fold and 1.38-fold higher than under R, BR and RB, respectively. *Cyclin D2* controls the promotion of cell division and the inhibition of cell differentiation (Wang *et al.* 2006). *CDK A2* plays a critical role in controlling the cell cycle (Park *et al.* 2016). *E2FE* has been shown to inhibit the endocycle (Sozzani *et al.* 2010). The expression of *EL2* under red light was higher than under other treatments, and *EL2* operates as a cell-cycle inhibitor (Peres *et al.* 2007). The relative expression of most DEGs was up-regulated under BR and R compared to RB and B, with the exception of *cyclin A2*, *CDK B2-1*, *cyclin D4*, *cyclin5*, *wee1* and *cdc6*. The differential expression of a total of 23 DEGs between light comparisons indicates that the cell cycle in the maize leaf is sensitive to blue and red light regulation.

### A total of 23 differentially expressed cell-cycle-related genes were annotated by gene ontology (GO) and Kyoto encyclopaedia of genes and genomes (KEGG)

**Figure 2.**
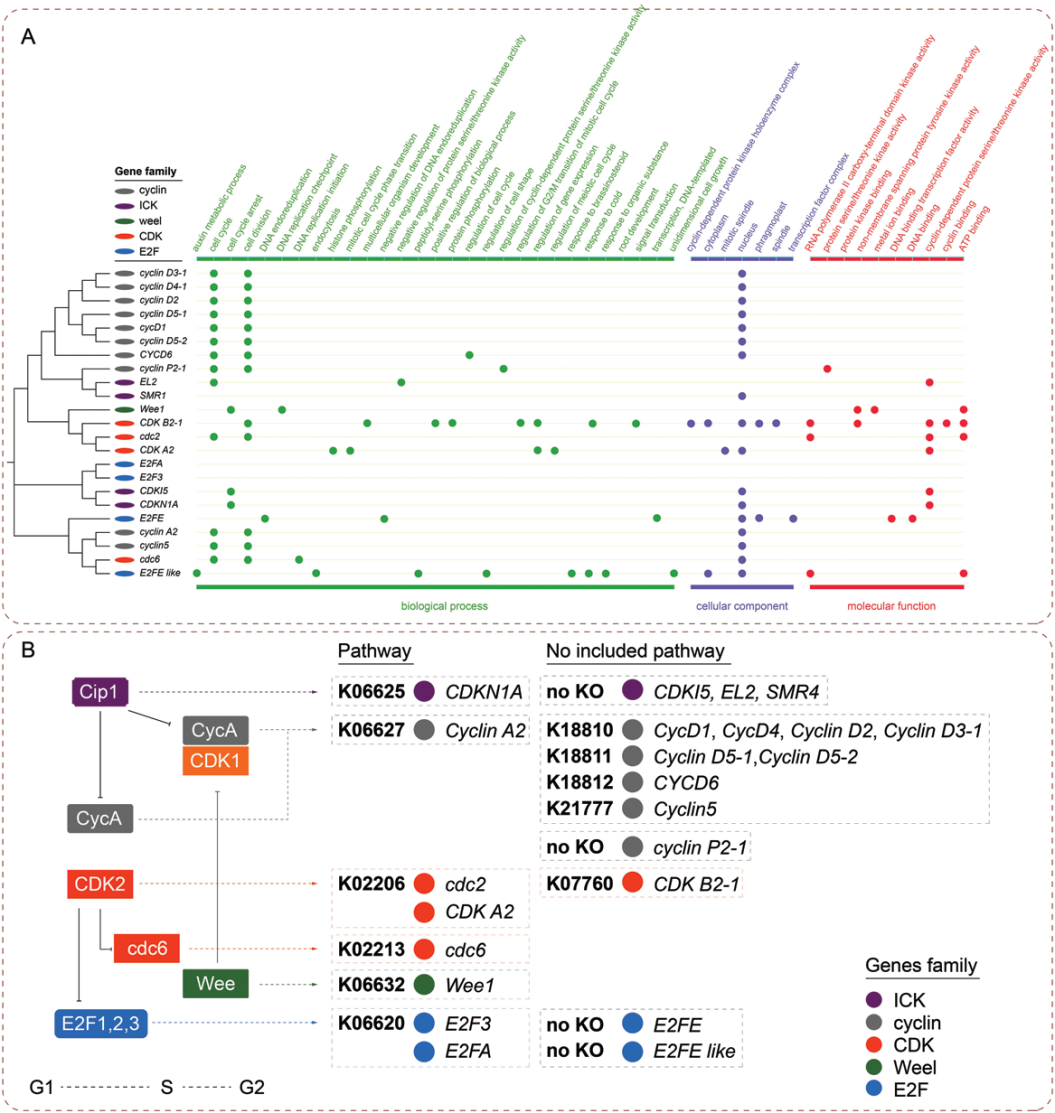
(A) Phylogenetic tree and GO functional annotation of 23 DEGs in cell cycle according to the NCBI and UniProt Database. (B) KEGG annotation of 23 DEGs in cell-cycle metabolic pathway.

The results of the transcriptome sequencing showed that 23 DEGs were enriched in the cell-cycle regulation pathway in response to blue, red and blue-red conversion light conditions. A phylogenetic tree was drawn to determine the neighbour-joining relationship of these 23 DEGs (Fig. 2A). The identity of the 23 DEGs was retrieved from NCBI and UniProt. Among them, 10 cyclin genes belong to three different classes: *cyclin D3-1*, *cyclin D4-1*, *cyclin D2*, *cyclinD5-1*, *cycD1*, *cyclin D5-2* and *CYCD6* belong to the cyclin delta class; *cyclin P2* belongs to the cyclin P class and *cyclin A2 and cyclin5* belong to the cyclin A class. Based on the difference and clustering of conserved protein domains, *EL2* is a new type of ICK gene, and *SMR1* belongs to the SIAMESE-RELATED family. *CDKI5* and *CDKN1A* belong to the ICK/KRP subfamily (PF02234, CDI) of the ICK gene family. *weel* is a gene in the weel family. *CDK B2-1* is a B2-type CDK gene, and *cdc2* (*CDKA;1*) and *CDK A-2* are homologues of mammalian Cdk1 and Cdk2 present in plants. *Cdc6* is a gene in the cdc18/cdc6 gene subfamily. *E2FA* and *E2F3* are the ‘activating’ subgroup of the E2F family (Attwooll *et al.* 2004). E2FE and E2FE-like are genes in the E2F-like family.

GO analysis was conducted to assess the specific functions of the 23 DEGs. The details of GO terms are described in Fig. 2A. According to the GO classification, 29, 7 and 10 terms were significantly enriched in three categories respectively: ‘biological process’, ‘cellular component’ and ‘molecular function’. Thirteen DEGs each were involved in the top two terms in ‘biological process’: ‘cell cycle’ and ‘cell division’. Seventeen DEGs were involved in the top term in ‘cellular component’: ‘nucleus’. Six, 4 and 3 DEGs were involved in the top three terms in ‘molecular function’: ‘cyclin-dependent protein serine/threonine kinase activity’, ‘ATP binding’, and ‘RNA polymerase II carboxy-terminal domain kinase activity’, respectively.

All 23 DEGs were annotated using KEGG to identify the metabolic pathways in which they function. The results show that eight DEGs are involved in the cell-cycle metabolic pathway in response to blue, red and blue-red conversion light (map04110) (Fig. 2B). One DEG, *CDKN1A*, was significantly enriched in ‘Cip1’. The other three ICK family DEGs (*CDKI5*, *EL2* and *SMR4*) were not annotated by KEGG ontology (KO). One of the nine DEGs in the cyclin family (*cyclin A*) could be annotated as CycA, but eight other cyclin family members could not be confirmed by KO in the cell-cycle metabolic pathway. Two of the eight DEGs (*cdc2* and *CDK A2*) could be annotated as *CDK2*. Annotated *CDK B2-1* does not participate in the cell-cycle metabolic pathway. *cdc6* was annotated as *cdc6*. *Wee1* could be annotated as *Wee*. *E2F3* and *E2FA* could be annotated as *E2F1*,*2*,*3*. *E2FE* and *E2FE-like* did not have any KO annotation.

### Discovery of unidentified potential functional genes in the gene interaction network of the cell cycle

**Figure 3.**
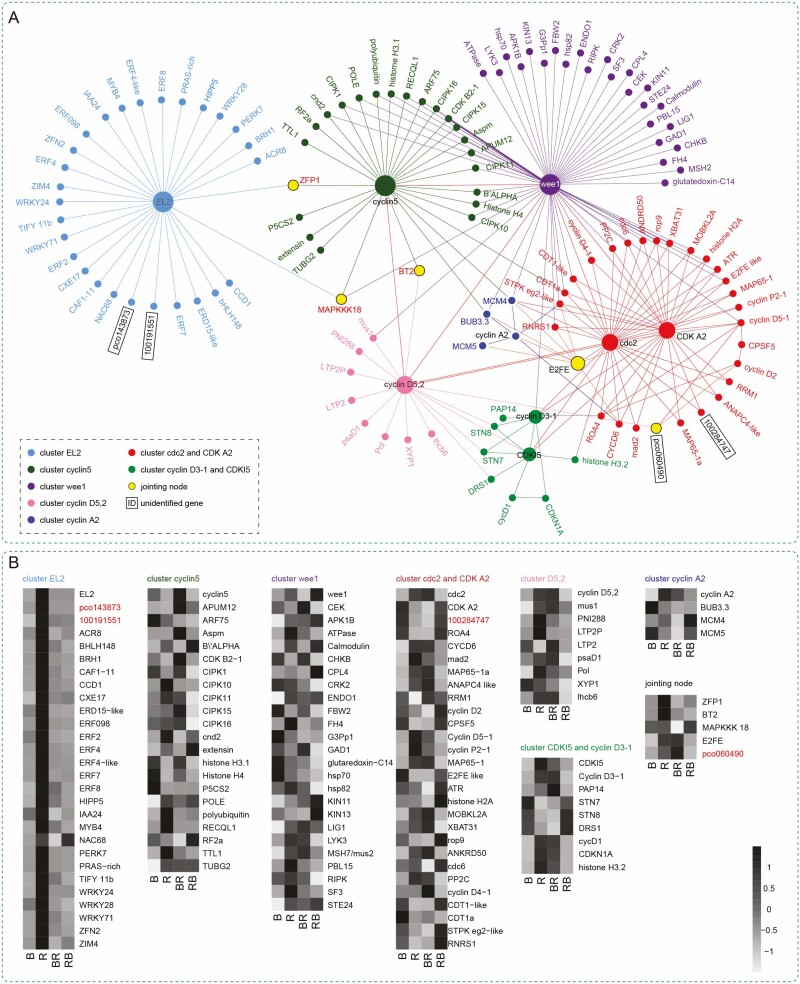
(A) Topological gene interaction network analysis using 23 cell cycle-related DEGs in total R-seq. (B) The RNA-seq expression of 130 DEGs within gene interaction network.

To find unidentified potential functional DEGs interacting with cell-cycle-related DEGs, we constructed a gene-interaction network using the MCODE app of Cytoscape software (Fig. 3A). An interaction network between 23 DEGs and all DEGs in the transcriptome was constructed to evaluate the potential interaction of the DEGs in response to the four light conditions. A total of 130 DEGs were characterized among all samples by analysing the normalized FPKM value of transcripts obtained from the transcriptome data. These 130 DEGs were classified into eight clusters by clustering centre. Seven of them were named as follows: cluster *EL2*, cluster *cyclin5*, cluster *Wee1*, cluster *cyclin D5,2*, cluster *cyclin A2*, cluster *cdc2* and *CDK A2*, and cluster *cyclin D3-1* and *CDKI5,* by centre gene name, respectively. An additional cluster was named the jointing node. In cluster *EL2*, 28 DEGs interacting with *EL2* were obtained. Two unidentified genes, *pco143873* (*LOC100280483*, *GRMZM2G355572*) and *1001191551* (*LOC100191551*, *GRMZM2G037015*), were discovered. The RNA-seq expression for each gene under the four light treatments is shown as normalized data (Fig. 3B). The expression patterns of the two unidentified genes were consistent with other DEGs in cluster *EL2* except for *NAC68*. In detail, the expression of DEGs under red light was significantly higher than under other treatments. In cluster *cdc2* and *CDK A2*, an unidentified gene *100284747* (*LOC100284747*, *GRMZM2G148621*) was obtained. The expression pattern of *100284747* was consistent with *CDK A2*, but not with *cdc2*. The expression of 100284747 was higher under B and RB light than under R and BR light. In the cluster jointing node, *pco060490* (*LOC100383344, GRMZM2G056093*) was found linking *cyclin D5*,*2* with *cyclin D5-1* and *cyclin D2*. Its expression was higher under R and BR than under B and RB light, which is consistent with *cyclin D5-1* and *cyclin D5*,*2*, but contrary to *cyclin D2*.

### Identification of potential function of four unidentified DEGs in cell-cycle signalling pathways

**Figure 4.**
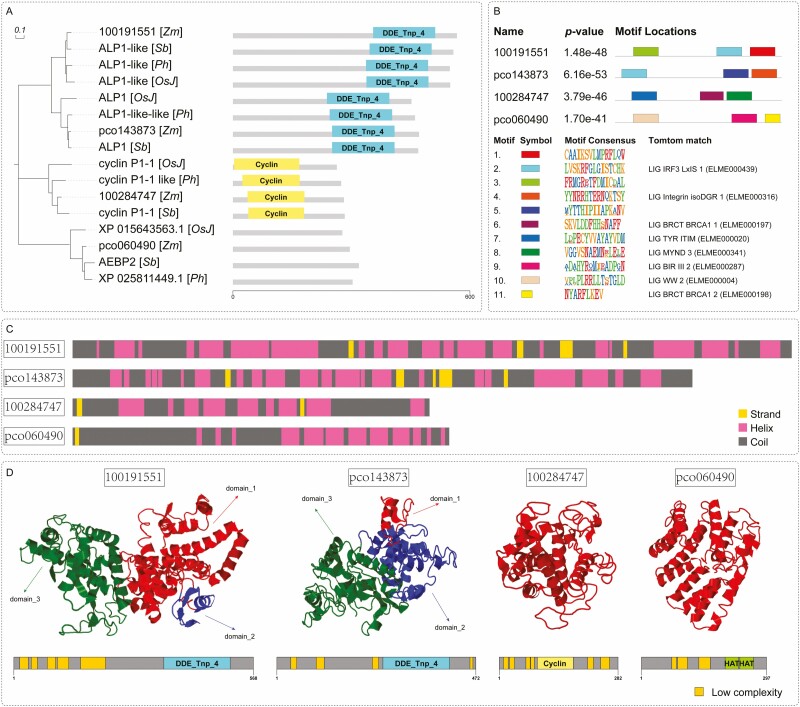
Homologous comparison of conserved domain (A) and motif (B) of four unidentified proteins compared with *Oryza sativa japonica* (*OsJ*), *Panicum hallii* (*Ph*), and *Sorghum bicolor* (*Sb*). Prediction of secondary (C, D) and tertiary (D) structure of four unidentified proteins: 100191551, pco143873, 100284747 and pco060490.

To further explore the functional relationship between four unidentified DEGs in three Gramineae plants, namely, *Oryza sativa japonica* (*OsJ*), *Panicum hallii* (*Ph*) and *Sorghum bicolor* (*Sb*), multiple sequence alignment using the Protein Basic Local Alignment Search Tool (BLASTP) was carried out to determine the neighbour-joining relationship of 16 genes (Fig. 4A). After mRNA was translated into proteins, the predicted protein domains of the four unidentified DEGs were used to indicate the difference from homologous proteins in other Gramineae plants. The phylogenetic relationship revealed that 100191551 was similar to ALP1-like, pco143873 was a neighbour of ALP1 (amphiphysin-like protein 1) and ALP1-like-like (ALP1-ll), 100284747 shared a branch with cyclin P1-1, and pco060490 was close to AEBP2 of *S. bicolor*. To further identify the domains of the four unidentified DEGs, Pfam was conducted to predict their conserved domain. 100191551 and pco143873 had a conserved domain of ‘DDE_Tnp_4’ which belongs to the ALP protein family. 100284747 had a conserved ‘cyclin’ domain, which is a cyclin protein domain in other Gramineae plants. Pfam analysis of pco060490 did not reveal any obvious conserved domain. MEME-ChIP tool was used to perform a comprehensive motif analysis of four unidentified proteins from the four unidentified DEGs. A total of 11 conserved motifs were discovered (Fig. 4B); eight of them were similar to known binding motifs, and three of them were mismatched in Tomtom with any eukaryotic linear motif resource (Fig. 4B).

Protein structure is directly correlated to biological function. After gene transcription, the amino acids form peptide chains and tertiary structures to exert their functions. To further define the structure of these four unidentified DEGs, protein sequence analysis was used to determine the secondary and tertiary structure (Fig. 4C and D). Meanwhile, the I-TASSER method was used to predict the structure of these four proteins. The confidence scores of the structures predicted were given as the eTM score to estimate the quality of the predicted models. In this study, the predicted results indicated that 100191551 consisted of 568 amino acids (aa). The secondary structure was composed of 22 strands, 287 helixes and 259 coils. The eTM-score for the three domains of 100191551 was 0.21 (aa, 1–185, 216–326), 0.56 (aa, 186–215) and 0.43 (aa, 327–568). pco143873 consisted of 472 aa. The secondary structure included 25 strands, 215 helixes and 232 coils. The eTM-score for three domains of pco143873 was 0.48 (aa, 1–35), 0.33 (aa, 36–221) and 0.38 (aa, 222–472). 100284747 consisted of 282 aa. Its secondary structure included 7 strands, 118 helixes and 157 coils. The confidence eTM-score for the predicted domains of 100284747 was 0.55. pco060490 consisted of 297 aa. Its secondary structure included 3 strands, 107 helixes and 187 coils. The confidence eTM-score for the predicted domains of pco060490 was 0.65. To predict the secondary structure of the four unidentified DEGs more precisely, the SMART tool was used to detect the PRO SPERO repeats, signal peptide, transmembrane domains, unstructured regions and low-complexity regions. Low complexity and HAT domains were found in four unidentified proteins. Five, 4 and 6 low-complexity sequence fragments were found in 100191551, pco143873 and 100284747, respectively. Three low complexity sequence fragments and two HAT SMART domains were found in pco060490 (Fig. 4D).

**Figure 5.**
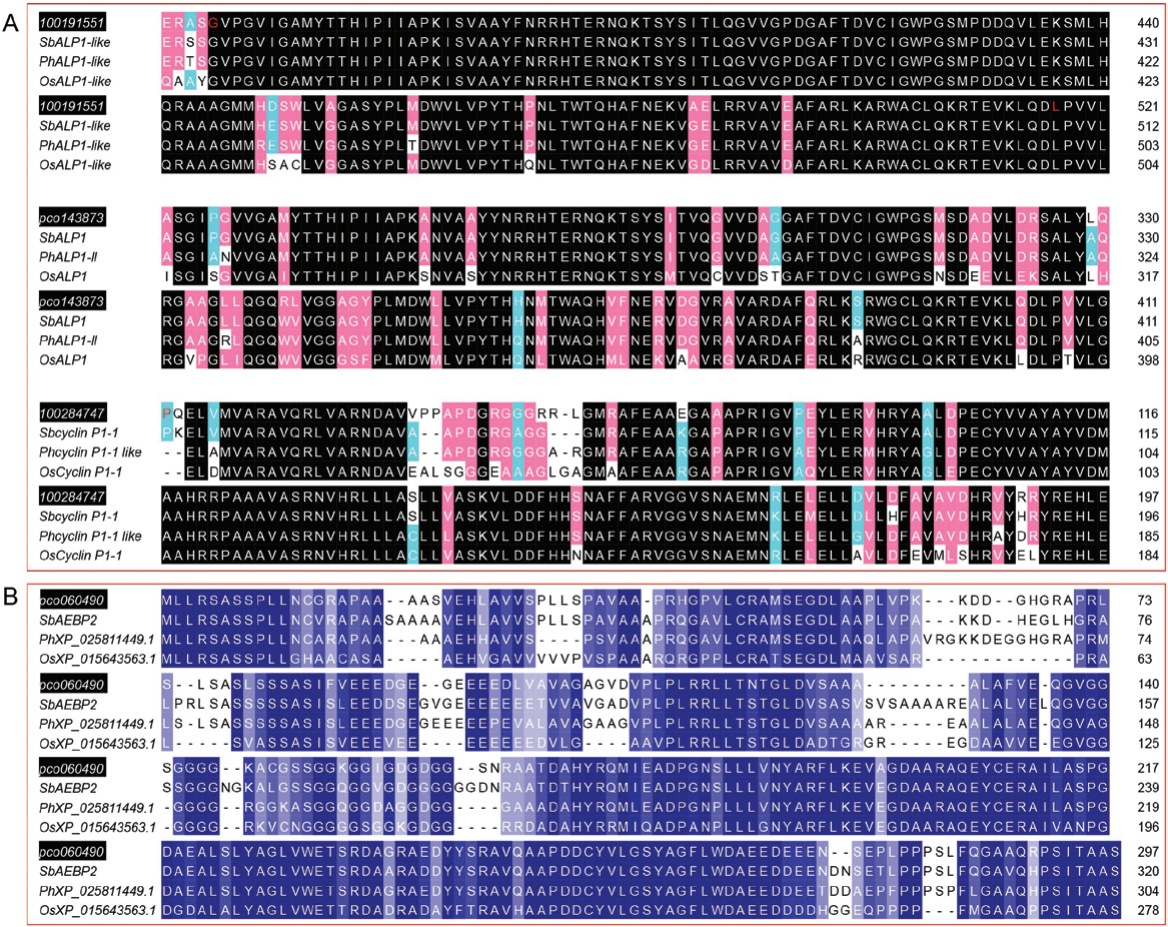
(A) Multiple sequence alignment profile of conserved domain of 10019551, pco143873 and 100284747 compared with *Sorghum bicolor*, *Panicum hallii* and *Oryza sativa japonica*. Black represents 100% sequence identity. Pink represents more than 75% while cyan represents less than 75% sequence identity. (B) Multiple sequence alignment profile of total sequence of pco060490 compared with *S. bicolor*, *P. hallii* and *O. sativa japonica*, the darker blue represents the highest identity.

The interspecies homology of the individual amino acids in the conserved region of 100191551, pco143873 and 100284747 was high (>70%) (Fig. 5A). A total of 144 of 154 aa in the conserved domain region of 100191551 were 100% identical when compared to three species of Gramineae. If only 100191551 and ALP1-like [*Sb*] were compared, there were three aa differences, indicating high homology (98%). A total of 123 of 162 aa in the conserved domain region of pco143873 were 100% identical when compared to three Gramineae plants. If pco143873 was only compared with ALP1 [*Sb*], homology reached 97.5% (3 aa difference). For 100284747, homologous matching decreased to 75% when multiple alignment was carried out among the four species, and the non-homologous matching rate was only 7.6% when 100284747 was compared with cyclin P1-1 [*Sb*]. A multiple sequence alignment profile of the total sequence of 10038334 was compared with *S. bicolor*, *P. hallii* and *O. sativa japonica* to identify the orthologues (Figure 5B). Among these species, pco060490 showed the highest homology with AEBP2 of *S. bicolor* (87.5%).

### Protein–protein interaction (PPI) network of cell-cycle-related genes that four unidentified DEGs connected

**Figure 6.**
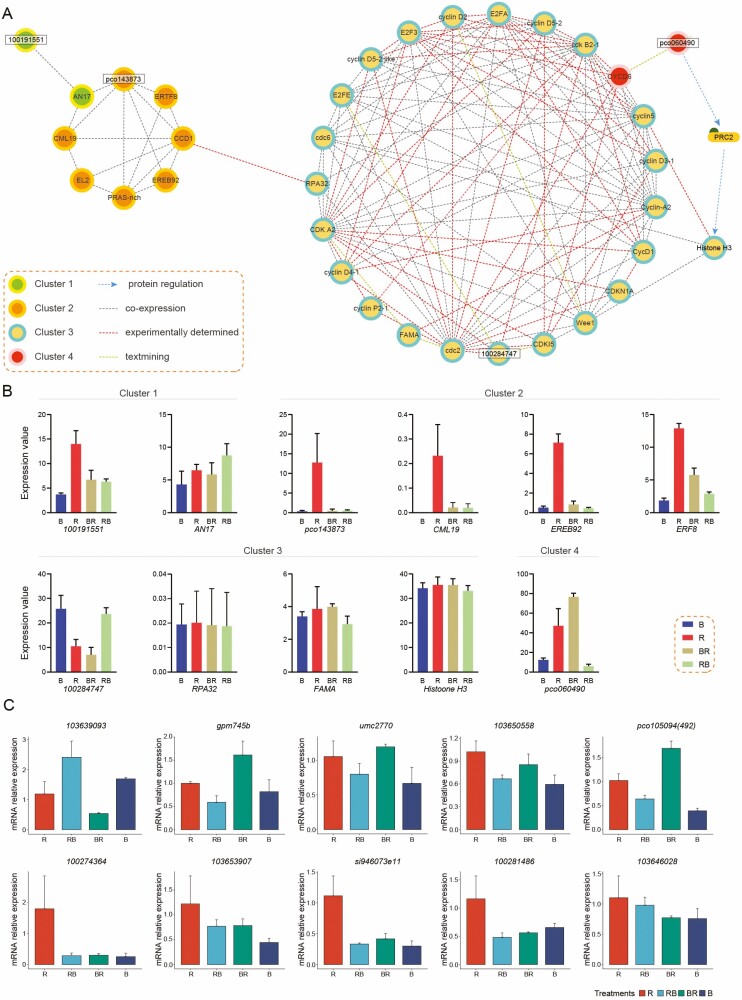
(A) PPI network of 35 proteins in cell-cycle functional pathway retrieved from STRING 11.0, and clustered by MCL method in inflation parameter 3. (B) The RNA-seq expression of four unidentified and seven directly related genes in the PPI of the cell cycle. (C) Ten DEGs randomly selected for qPCR validation.

To gain further insight into the function of four unidentified genes in the cell-cycle signal network, the PPI network composed of 35 proteins was depicted using the String online tool (Fig. 6A) with the Markov Cluster (MCL) clustering method, for which the inflation parameter was set to 3. Four clusters were obtained, and seven directly related proteins (AN17 (*LOC100127535*), CML19 (*LOC103636275*), ERF8 (*LOC103630553*), EREB92 (*LOC100280582*), RPA32 (*LOC100272286*), FAMA (*LOC103635409*) and Histone H3 (*LOC103627323*)) joined the cell-cycle PPI network. The expression levels of the above-related DEGs were plotted (Fig. 6B). In cluster 1, 100191551 co-expressed with AN17, which is a ZnF-AN1 family protein (Jin *et al.* 2007). In cluster 2, a co-expression network was built from unidentified protein pco143873 and five reported proteins, which directly co-expressed with pco143873. Among them, CML19 is a calmodulin-like protein that modulated nucleotide excision repair by binding to RAD4 protein in response to different external stimuli by Ca^2+^ second messenger in *Arabidopsis* (La Verde *et al.* 2018). EREB92 is also known as ethylene-responsive transcription factor 4, which is a transcriptional repressor capable of modulating ethylene and abscisic acid responses (Yang *et al.* 2005), and negatively regulated the iron deficiency response in *Arabidopsis* (Liu *et al.* 2017). Ethylene-responsive transcription factor 8 (ERTF8) plays a positive role in mediating immunity and programmed cell death (Cao *et al.* 2018). EF-hand Ca^2+^-binding protein CCD1 (*LOC100285110*) positively regulates osmotic and salt tolerance in rice (Jing *et al.* 2016). Therefore, pco143873 and 110191551 show a potential responsivity to abiotic stress. Additionally, the function of PRAS-rich protein (*LOC100285353*) remains unknown. The expression levels of the four DEGs in cluster 2 were significantly higher under red light than under the other treatments. In cluster 3, 100284747 interacted with CDK A2, cdc2 and CDK B2-1 in the ‘experimentally determined’ type of interaction, which indicated that 100284747 may be involved in the regulation of cell cycle division. In cluster 4, pco060490 only interacted with CYCD6 in the ‘text-mining’ type of interaction, and there is no experimental evidence to prove its protein association. However, after expanding the PPI network, we established the protein interaction that linked pco060490 with cell-cycle-related proteins by Histone H3 and Polycomb Repressive Complex 2 (PRC2). PRC2 is a transcriptional repressor complex best known as a ‘writer’ of H3K27 methylation in histone H3, a modification associated with epigenetic gene silencing. This complex plays a fundamental role in regulating cellular differentiation and development (Moritz and Trievel 2018). To validate the accuracy of the transcriptome data presented in this study, a random selection of 10 DEGs underwent qPCR expression analysis. The results demonstrate that the transcriptome sequencing data in this article are reliable (Fig. 6C).

## Discussion

Maize is a typical high-efficiency light-harvesting crop, and its cell development and differentiation are very sensitive to light (Liu *et al.* 2019, 2020). Cell division in photosynthetic organisms is tightly regulated by light. Light-dependent signal transduction leads to great differences in the RNA-seq expression of cell-cycle-related genes. Blue and red light significantly mediate the expression of cell-cycle-related genes, and thus affect specific cell initiation. The blue light checkpoint is the key to controlling the onset of cell division (Lockhart 2013). Blue light induces *cyclin2* expression during the cell cycle. The product of cyclin2 binds to the cyclin-dependent kinase (CDKA1) and can complement G1 cyclin-deficient yeast (Huysman *et al.* 2013a). But blue light has also been reported to induce cell-cycle arrest by downregulating CDKN1B and reactive oxygen species (Becker *et al.* 2016). Red light promotes high growth rates but induces a smaller cell size by accelerating the cell cycle in some microalgal species (Schulze *et al.* 2014). In this study, 23 differentially expressed genes were enriched in the ‘cell cycle’. The top three of the second level of GO annotation were mainly enriched in terms of ‘cell cycle’, ‘cell division’ and ‘nucleus’. KEGG analysis revealed that the light signal mainly mediates the G1/G2 phase in the cell cycle of maize. Specifically, active and overexpressed CDKN1A increased cell cycle arrest in the G1 phase (Russo *et al.* 1996). The down-regulation of cyclin A2 and CDK2 (cdc2 and CDK A2) is also involved in cell-cycle arrest (Habib 2010). E2F3 and E2FA are the central regulators of the cell cycle; they bind target promoters and recruit additional activating proteins to stimulate the expression of cell-cycle genes during G1/S (Roelofs *et al.* 2020). The Cdc6 protein is localized in the cell nucleus during the G1 phase of the cell cycle and plays key roles in regulating DNA replication and the activation and maintenance of cell-cycle check-points (Youn *et al.* 2020). Once cdc6 disappears, DNA duplication no longer occurs during the cell cycle (Kurata *et al.* 2003). Wee1, as a negative regulator of CDKs, inhibits mitotic exit, regulates the temporality of cell-cycle progression, and also impacts cell proliferation (Kratassiouk *et al.* 2016). Different light conditions regulate the cell cycle by light-dependent photolyase activity (Zhao and Mu 1998; Liu and Zhang 2021a), meanwhile, DNA damage delays cell-cycle entry by affecting cell cycle checkpoints, causing cell-cycle arrest at specific stages (Li *et al.* 2017). In this study, the RNA-seq expression of the above-mentioned genes depicted the advantage of blue light over red light in terms of cell-cycle mediation. To some extent, the above processes explain the difference in the specialized cell formation during cell cycles under red light in maize (Liu *et al.* 2020; Liu and Zhang 2021a).

Comparative transcriptome analysis can be used to identify essential genes involved in specific metabolic pathways (Liu and Zhang 2021a, 2021b). In this study, four unidentified genes were found in six comparisons between light treatments. Through multiple alignment analyses of the sequences and conserved domains, the characteristics of these four unidentified proteins were preliminarily determined. A probable ALP1-like (100191551) and a probable ALP1 (pco143873) protein were obtained after protein structure analysis. APL1 is the most abundant and possibly most essential element for evolution in viral, bacterial and eukaryotic genomes. It can fulfil essential functions for an organism, such as DNA processing (Nowacki *et al.* 2009). APL1 encodes a gene related to *Harbinger*-like transposases, which features an endonuclease domain of the DDE-4 superfamily, these endonucleases contain a catalytic triad of three acidic amino acid residues that coordinate metal ions needed for catalysis (Hartwig *et al.* 2012). The APL1 interacts functionally with the cABL tyrosine kinase and may play a role in cytoskeletal regulation (Kadlec and Pendergast 1997). CyclinP1-1 (CYCP1;1) was equally detected in all plant tissues, as a PHO8o homologous protein, and negatively regulated phosphate starvation signalling in the roots of rice and *Arabidopsis* (Torres Acosta *et al.* 2004; Deng *et al.* 2014). In this study, we found a probable cyclin P1-1 protein (pco060490) expressed differentially in maize leaf, but its function needs further study. pco060490 was identified as a probable AEBP2 protein, which encodes an evolutionarily conserved zinc finger protein that is closely associated with the Polycomb Repressive Complex 2 (PRC2). AEBP2 has a stabilizing effect on monomeric PRC2 and inhibits the formation of PRC2 dimers (Chen *et al.* 2020). In plants, PRC2 catalyzes the trimethylation of Histone 3 protein at the lysine 27 position (H3K27me3), the hallmark of a silent chromatin state that is correlated with gene repression and self-maintenance across cell division (de Lucas *et al.* 2016). Finally, PRC2 exerts repressive functions on negative regulators of the cell cycle (Crippa *et al.* 2016). Therefore, the function of AEBP2 in the cell-cycle process is complex.

Based on the homologous alignment of four unidentified protein sequences, we also predicted their probable signal positions in the PPI network using the STRING database. In this depicted PPI network, 100191551 is co-expressed with AN17, which is a gene in the ZnF-AN1 family that is reported to be involved in responding to abiotic stress. In maize, northern blot analysis showed that *ZmAN17* was elicited by cold stress (Jin *et al.* 2007). Pco143873 is co-expressed with CML19, which has been reported as a factor responding to environmental stress (La Verde *et al.* 2018); its function is related to mediating the immunity factor ERTF8 and abiotic stress response factors EREB92 and CCD1 (Yang *et al.* 2005; Jing *et al.* 2016). Therefore, we concluded that 100191551 and Pco143873 are potentially regulated by abiotic stress response factors to avoid DNA damage during the cell cycle. The existing experimental evidence determined the locations of cyclin P1-1 (100284747), CDKs (CDK A2, CDK B2-1, cdc2) and CDKI (CDKI5). We inferred that cyclin P1-1 negatively regulates the cell cycle in response to phosphate starvation signalling (Lenburg and O’Shea 1996). For AEBP2, according to the expression level of CYCD6 and Histone H3, the cell cycle under red light may be inhibited by the more active CYCD6 and Histone H3. Histone H3 is a cell-cycle marker, and phosphor-Histone H3 increases cell-cycle re-entry. Histone H3 acetylation leads to G0/G1 cell-cycle arrest (Laurenzana *et al.* 2013). Considering the stability of the expression of CYCD6 and Histone H3, there may be other cascades that work with AEBP2.

In conclusion, blue and red light independently affect the cell cycle in maize leaves by known and unknown factors. Through comparative transcriptome analysis of cell-cycle-related DEGs, four novel unidentified potentially functional genes were found. The results of this study should yield further insight into the cell-cycle mechanism of maize.

## Supplementary Material

plad079_suppl_Supplementary_Tables_S1

## Data Availability

The datasets used and/or analysed during the current study are available from the corresponding author on reasonable request. Or these data can be accessed in NCBI SRA database (https://www.ncbi.nlm.nih.gov/Traces/study/?) by PRJNA743131.
