# Supplementary material for: Transcriptome-based network analysis of cell cycle-related genes in response to blue and red light in maize
Source: AoB Plants. 2023 Dec 11;15(6):plad079. doi: 10.1093/aobpla/plad079 (PMC10712220; doi:10.1093/aobpla/plad079)
Supplement: plad079_suppl_Supplementary_Tables_S1 [file plad079_suppl_supplementary_tables_s1.docx]

| Gene name | Primer sequences |
| --- | --- |
| 103639093-F  103639093-R | CATCACCGACAACGACAACT  CGCATCTCCACCTTCTTCAG |
| gpm745b-F  gpm745b-R | TCTCCATTCCACCACTATCTGTAG  CGCTTGGATATGCTGTTCTTCA |
| umc2770-F  umc2770-R | CAGTACAGACAGTTGGAGTTCAG  AGGCGGCTTAGATGTGATGT |
| 103650558-F  103650558-R | CTACTGACAGAGGCGAGCAT  TTCTGGAACTCAGCGAAGGA |
| pco105094(492)-F  pco105094(492)-R | GCTGAGGATGCCATTATGCTA  GACAGACTAATGCGTCGTTCA |
| 100274364-F  100274364-R | ACAACCTGAGCGATGAGGAG  TCGTACACCAGGTAGTGGCA |
| 103653907-F  103653907-R | TCTCGACCGCACGATTGAAC  ATGGAGCACTGGGCATCATC |
| si946073e11-F  si946073e11-R | AGGCTTGTTGCGACTGAACT  ACAATTGACTCCCGAGCGTG |
| 100281486-F  100281486-R | AGCTTCGGCCTCAGCATCCTCG  GGACGGTTCACCGGGTTCTTCTGTA |
| 103646082-F  103646082-R | TTCACATACGAAGCAATACATCCT  GCACCATTACCACCAGATAGG |
| GADPH-F  GDAPH-R | TACTGTGGATGTCTCGGTTGT  CTGCTGTCACCAAGGAAGTC |
